# Supplementary material for: Multiple Dirac cones at the surface of the topological metal LaBi
Source: Nat Commun. 2017 Jan 9;8:13942. doi: 10.1038/ncomms13942 (PMC5227739; doi:10.1038/ncomms13942)
Supplement: Supplementary Information — Supplementary Figures [file ncomms13942-s1.pdf]

Supplementary Figures

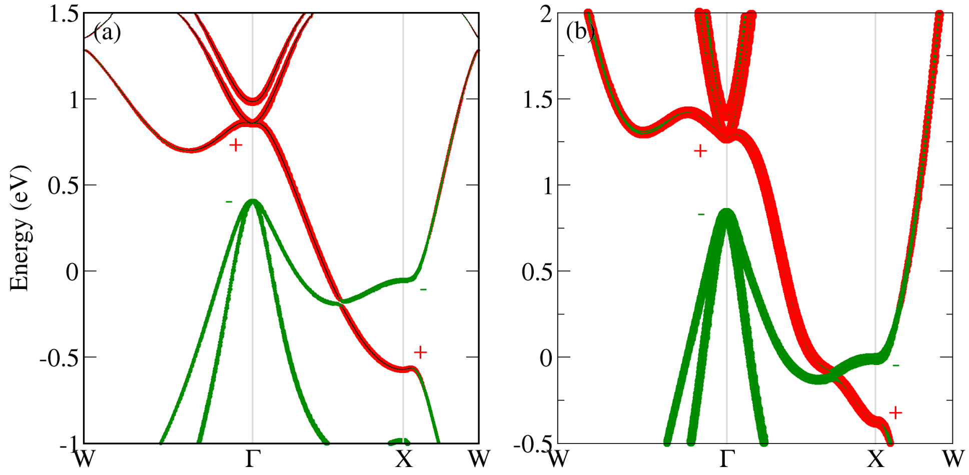

Supplementary Figure 1: The bulk band structure of LaBi. (a) Calculations within the generalized-gradient approximation (GGA) level. **(b)** Calculations in the hybrid-functional (HSE06) level. The Fermi energy is shifted to zero. The red and green dots represent the orbital contribution from La-d (parity +) and Bi-p (parity –) states to the band structure, , respectively. It is clear that La-d and Bi-p bands get inverted between Γ and X points. The parity eigen values (+ or –) are specified for the conduction and valence bands. Since GGA is known to usually overestimate the band inversion strength, we performed HSE06 calculations that correct the GGA error, to validate the inverted band structure. It is clear that the topological band inversion occurs for both GGA and HSE06 calculations, resulting nontrivial Z_2 index $\nu_0 = 1$ based on the parity criteria.

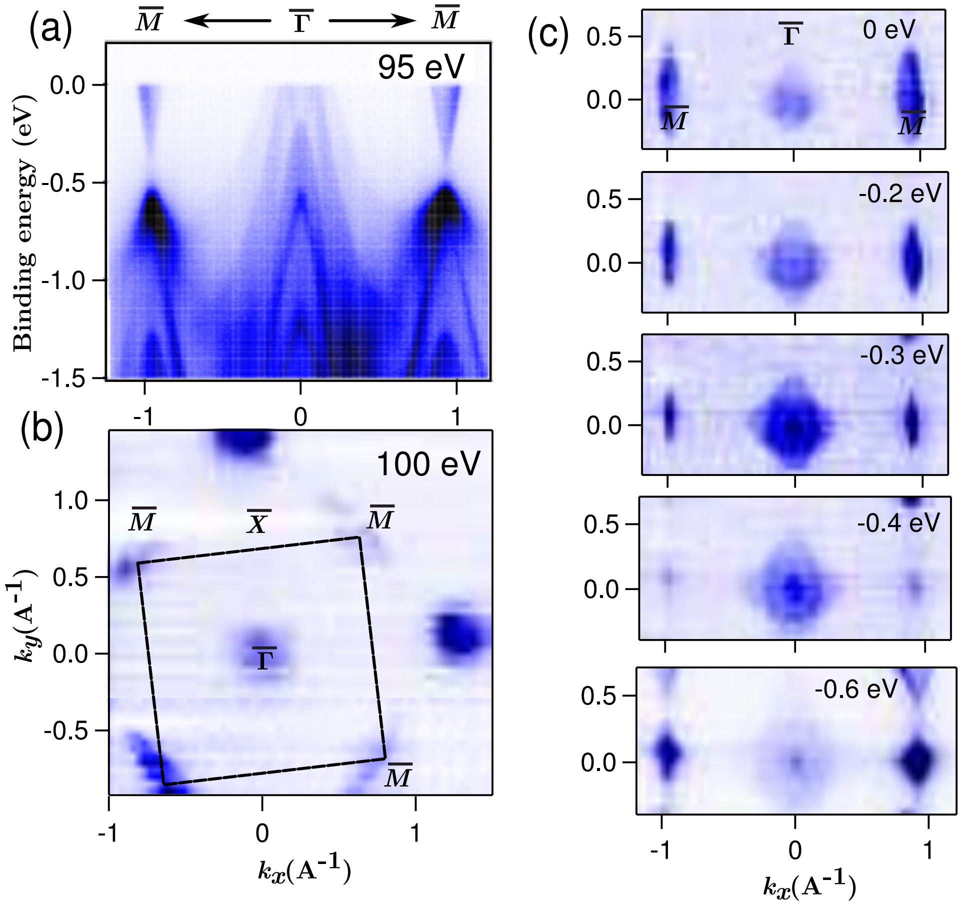

Supplementary Figure 2: The ARPES measurement on the GdSb (001) surface. (a) The band dispersion along the [100] direction ($\overline{M}$-$\overline{\Gamma}$-$\overline{M}$) measured with a photon energy of 95 eV. About 0.38 eV below the Fermi energy, an energy gap of 0.16 eV is observed at $\overline{M}$ between upper and lower topologically trivial bands. There is no apparent Dirac points at $\overline{M}$ and $\overline{\Gamma}$ points. **(b)** The Fermi surface measured with a photon energy of 100 eV. **(c)** The Fermi surface at different Fermi energies. We note that GdSb which posses the same crystal structure as LaBi, but exhibits a G-type antiferromagnetic phase below 20 K. ARPES was measured at a temperature of 1 K. From the surface states, we can conclude that GdSb is topologically trivial, distinct from LaBi.

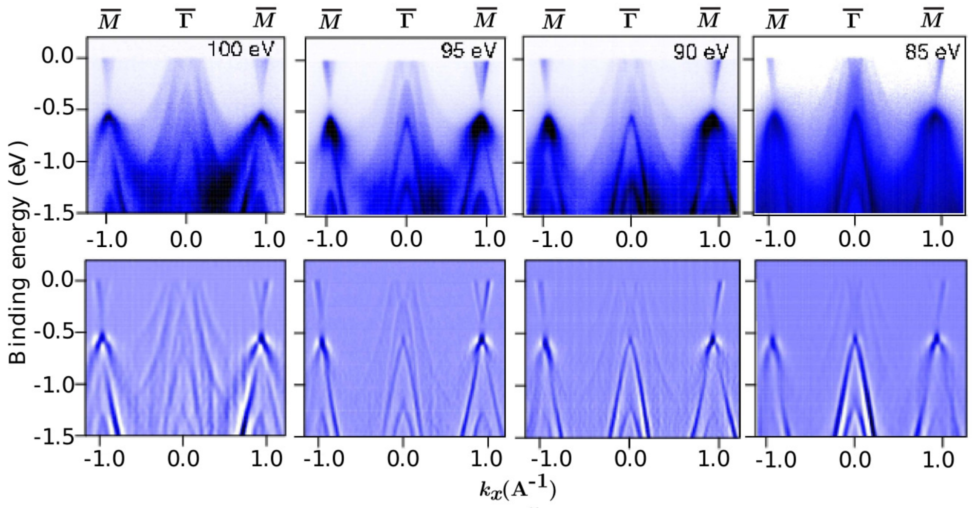

Supplementary Figure 3: The band structure of GdSb measured with different photon energies. The upper panels show the photon energy dependent ARPES spectra and the lower panels show corresponding second derivatives of the spectra to make details better visible. It is clearly revealed, moreover, that no Dirac cone is detectable at the $\overline{\Gamma}$ and $\overline{M}$ points, in contrast to LaBi(001).

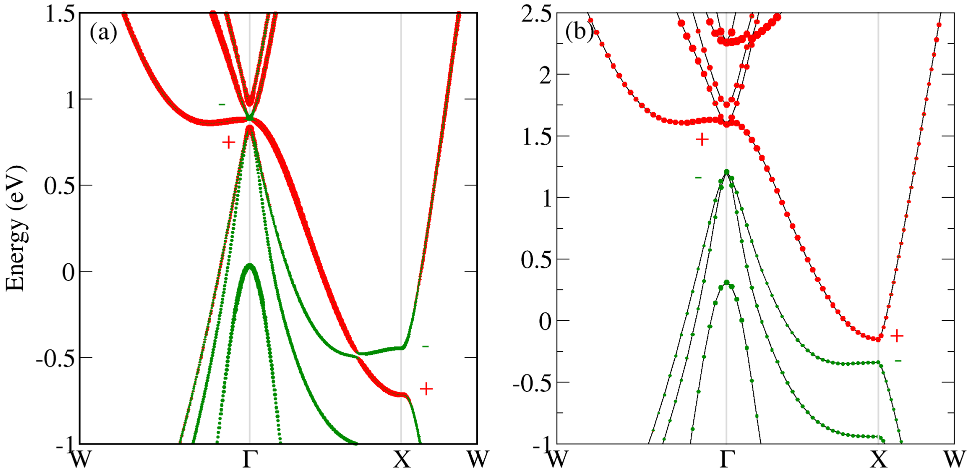

Supplementary Figure 4: The bulk band structure of GdSb. (a) Calculations within GGA. **(b)** Calculations within HSE06. The Fermi energy is shifted to zero. The Gd-f electrons are frozen into the core levels in the calculation. The red and green dots represent the orbital contribution from Gd-d (parity +) and Sb-p (parity –) states to the band structure, respectively. In the GGA band structure, the valence band maximum (VBM) and conduction valence minimum (CBM) are Sb-p states and Gd-d states, respectively, at both Γ and X points. Thus, the Z_2 topological invariant $\nu_0 = 0$ is trivial. In the HSE06 band structure, VBM and CBM are Gd-d states and Sb-p states, respectively, at both Γ and X points. Since the VBM and CBM are inverted at both Γ and X, the Z_2 invariant $\nu_0 = 0$ is still trivial. Therefore, theoretical bulk band structures indicate that GdSb is topologically trivial, which is consistent with ARPES.
